# Pulmonary epithelioid hemangioendothelioma misdiagnosed as a benign nodule

**DOI:** 10.1186/s12957-015-0518-5

**Published:** 2015-03-14

**Authors:** Minah Kim, Jinsun Chang, Hayoung Choi, In-Jae Oh, Chul-Kyu Park, Young-Chul Kim, Yoo-Duk Choi, Ju-Sik Yun, Sang-Yun Song, Kook-Joo Na

**Affiliations:** Department of Internal Medicine, Chonnam National University Medical School, 671 Jebong-ro, Dong-gu, Gwangju, 501-757 Republic of Korea; Lung and Esophageal Cancer Clinic, Chonnam National University Hwasun Hospital, 322 Seoyang-ro, Hwasun, Jeonnam 519-809 Republic of Korea; Department of Pathology, Chonnam National University Medical School, 671 Jebong-ro, Dong-gu, Gwangju, 501-757 Republic of Korea; Department of Thoracic and Cardiovascular Surgery, Chonnam National University Medical School, 671 Jebong-ro, Dong-gu, Gwangju, 501-757 Republic of Korea

**Keywords:** Epithelioid hemangioendothelioma, Multiple pulmonary nodules, Surgery

## Abstract

Pulmonary epithelioid hemangioendothelioma (PEH) is a rare vascular tumor of borderline malignancy that originates from endothelial cells. Chest computed tomography (CT) performed during a routine cancer screening revealed multiple small pulmonary nodules in a 50-year-old man who had previously undergone endoscopic submucosal dissection of early gastric cancer. To rule out metastatic nodules, a wedge resection of the left upper lobe was performed and the frozen biopsy reported a benign fibrotic nodule. Using immunohistochemistry, the final pathology was indicated to be PEH, and consecutive surgery for the right-side nodules was planned and performed.

## Background

Epithelioid hemangioendothelioma is a rare vascular tumor, originating from endothelial cells, which is histologically characterized by an epithelioid appearance [1]. The term was initially applied by Weiss and Enzinger to a soft tissue vascular tumor of borderline malignancy [2]. Pulmonary epithelioid hemangioendothelioma (PEH) is the currently preferred term for the neoplastic process, which was originally described as intravascular bronchioloalveolar tumor in the lung. Being considered a low to intermediate grade sarcoma, the tumor predominantly involves the liver, lungs, soft tissues, and rarely bones, and can be aggressive and multicentric, even resulting in systemic metastasis [3-5]. PEH typically occurs as bilateral multiple nodules among young women and has a variable clinical course [6].

We describe a case of PEH misdiagnosed as a benign fibrotic nodule from frozen biopsy analysis following video-assisted thoracoscopic surgery (VATS). We performed a consecutive operation for the other nodules after confirming PEH based on the permanent biopsy report.

## Case presentation

**Figure 1:**
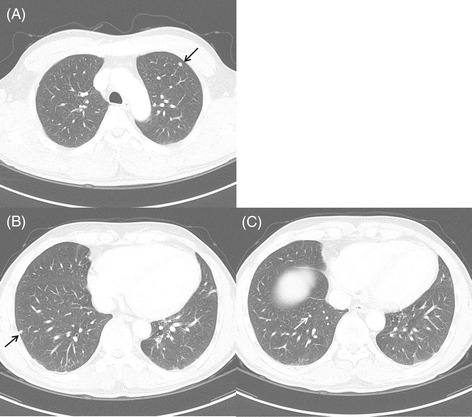
**The initial chest computed tomography scan. (A)** There is a peripheral small nodule with lobulation in the apicoposterior segment of the left upper lobe (arrow). **(B, C)** Two tiny nodules are noted in the posterobasal segment of the right lower lobe (arrows).

A 50-year-old man who visited the hospital every year after endoscopic submucosal dissection of early gastric cancer underwent a chest computed tomography (CT) for cancer screening. The patient was a 50-pack-year current smoker, but he did not have any respiratory or systemic symptoms. The chest CT scan showed small nodules on both sides of the lungs. There was a peripherally located small nodule with lobulation in the apicoposterior segment of the left upper lobe (LUL) and two tiny nodules in the posterobasal segment of the right lower lobe (RLL) (Figure 1). Levels of tumor markers, such as carcinoembryonic antigen and cancer antigen 19-9, were within the normal range. The preoperative forced expiratory volume in 1 s was 3.02 L (80% of the predicted value) without an obstructive pattern. Given that the patient was a current smoker, was older than 50 years, and had a gastric cancer history, we decided to perform a surgical biopsy of the nodules.

Under general anesthesia, the surgeons performed a wedge resection by VATS. The LUL nodule was palpable to the finger and was 1 cm in size, hard, and capsulated in nature. There was no adhesion with the lung parenchyma or pleura. The frozen biopsy showed a hypocellular nodule in a fibrotic stroma. It was reported as a fibrotic nodule. The RLL nodules were also considered as benign because of similar features. A CT follow-up of these nodules was planned rather than surgical resection. Postoperatively, the patient did not show any surgical complications.

**Figure 2:**
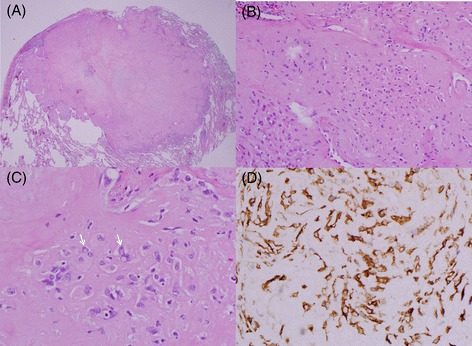
**Pathological findings. (A)** The nodule is well-demarcated, hyalinized, and within the lung parenchyma (hematoxylin and eosin, ×40). **(B)** Variable-sized and irregularly shaped tumor cells with round to oval shape nucleus are located in the hyalinized stroma. Some of the neoplastic cells are rich in eosinophilic cytoplasm (×200). **(C)** There are characteristic intracytoplasmic vacuoles in the tumor cells (arrow; ×400). **(D)** The tumor cells are immunoreactive for CD31 in the cytoplasm (×400).

The pathological findings of the resected LUL nodule indicated a well-demarcated hypocellular hyalinized nodule. Generally, the neoplastic cells were loosely embedded in hyalinized fibrous stroma individually, although it was more dense cellularly at the periphery of the nodule. The neoplastic cells showed no nuclear atypia and contained variably prominent eosinophilic cytoplasm with or without cytoplasmic vacuoles which was reminiscent of endothelial differentiation. The tumor cells also showed immunoreactivity for CD31 (Figure 2). These features are consistent with the histological characteristics of epithelioid hemangioendothelioma [7,8]. We decided to perform consecutive surgery of the two remnant tiny RLL nodules. Given that the preferred locations of epithelioid hemangioendothelioma are the liver and soft tissue, we performed an abdominopelvic CT before the second operation. There was no definite focal wall thickening and no enhanced masses were detected in the intraabdominal organs. The second operation was performed successfully using VATS; the pathological findings of the two tiny nodules were similar to those of the previous LUL nodule. After the second surgery, we performed follow-up chest CTs at 6-month intervals. There has been no evidence of recurrence or other metachronous nodules for 18 months.

## Discussion

Epithelioid hemangioendothelioma is a rare tumor, which mainly affects the liver and, more rarely, the lung. The other organs affected tend to be superficial or deep soft tissues of the extremities. The estimated prevalence of epithelioid hemangioendothelioma is less than 1 in 1 million [9]. Initially known as intravascular bronchioloalveolar tumor [4], PEH is a rare vascular tumor of an indeterminate or low-grade malignancy. PEH affects young people; the median age of onset is 36 years [10]. The incidence is two times higher in women than that in men. Approximately 50% to 76% of patients are asymptomatic. Some patients have chest pain, pleuritic pain, cough, dyspnea, or rarely hemoptysis. Although the typical CT findings are multiple small unilateral (23.7%) or bilateral (76.2%) pulmonary nodules, PEH can also present as multiple pulmonary reticulonodular opacities or diffuse infiltrative pleural thickening. Diagnosis is mainly achieved by pathological examination of the surgical biopsy and immunohistochemistry indicating diffuse factor VIII-related antigen cytoplasmic staining in the malignant cells, confirming an endothelial lineage for the tumor cells [7,11]. The other cell markers are CD31 and CD34 [12]. Owing to the low incidence of PEH, there are no definite treatment guidelines. The prognosis of this tumor is unpredictable, with life expectancy ranging from 1 to 15 years [5]. The presence of metastatic lesions at the time of diagnosis does not correspond with a reduced survival [7]. However, pleural effusion has been shown to correlate with a poor survival [12]. Due to the unpredictable nature of the outcome, if possible, curative resection should be considered to maximize the possibility of a good outcome. Adjuvant radiotherapy is used to control residual disease for patients with localized epithelioid hemangioendothelioma. Chemotherapy with interferon-2α or carboplatin plus etoposide is the preferred therapy for patients with widespread disease, but the benefits are unclear [4,13,14].

Our patient’s age was 50 years, which is somewhat older than the age at which patients typically present with PEH. He had bilateral pulmonary nodules and showed lobulated lesions without pleural thickening. Given the multiple small round pulmonary nodules, we considered metastatic nodules to be a possibility. From the results of the frozen biopsy analysis, we did not deem it necessary to resect all of the nodules; however, the final pathologic finding was indicated to be a PEH, consecutive surgery was performed.

## Conclusions

In conclusion, this case of PEH indicates the difficulty in diagnosing this rare tumor. Clinicians should be aware of the possibility of a low-grade malignancy, even if surgical frozen biopsy analysis indicates benign features.

## Consent

The study was performed in accordance with the Declaration of Helsinki and Good Clinical Practice guidelines. The study was approved by the Institutional Review Board of Chonnam National University Hwasun Hospital. Written informed consent was obtained from the patient for publication of this case and for the accompanying images.

## Abbreviations

CT: computed tomography
LUL: left upper lobe
PEH: pulmonary epithelioid hemangioendothelioma
RLL: right lower lobe
VATS: video-assisted thoracoscopic surgery
